# Neural theta oscillations support semantic memory retrieval

**DOI:** 10.1038/s41598-019-53813-y

**Published:** 2019-11-27

**Authors:** Martin Marko, Barbora Cimrová, Igor Riečanský

**Affiliations:** 1 Department of Behavioural Neuroscience, Institute of Normal and Pathological Physiology, Centre of Experimental Medicine, Slovak Academy of Sciences, Bratislava, Slovakia; 2 Department of Applied Informatics, Faculty of Mathematics, Physics and Informatics, Comenius University, Bratislava, Slovakia; 3 Social, Cognitive and Affective Neuroscience Unit, Department of Basic Psychological Research and Research Methods, Faculty of Psychology, University of Vienna, Vienna, Austria

**Keywords:** Cognitive control, Language, Long-term memory, Human behaviour

## Abstract

Lexical–semantic retrieval emerges through the interactions of distributed prefrontal and perisylvian brain networks. Growing evidence suggests that synchronous theta band neural oscillations might play a role in this process, yet, their functional significance remains elusive. Here, we used transcranial alternating current stimulation to induce exogenous theta oscillations at 6 Hz (θ-tACS) over left prefrontal and posterior perisylvian cortex with a 180° (*anti-phase*) and 0° (*in-phase*) relative phase difference while participants performed automatic and controlled retrieval tasks. We demonstrate that θ-tACS significantly modulated the retrieval performance and its effects were both task- and phase-specific: the *in-phase* tACS impaired controlled retrieval, whereas the *anti-phase* tACS improved controlled but impaired automatic retrieval. These findings indicate that theta band oscillatory brain activity supports binding of semantically related representations via a phase-dependent modulation of semantic activation or maintenance.

## Introduction

Diverse range of human behavior is semantically imbued, i.e., shaped and supported by a set of neurocognitive mechanisms that are collectively referred to as semantic cognition. Decades of research indicate that semantic cognition relies on two principal interacting cognitive and neural systems^1–3^. First is the system of semantic representation, which encodes multimodal knowledge structures involving sensory, motor, affective and linguistic information. Representations stored in this system can be effortlessly activated in face of external and internal cues (i.e., an automatic, stimulus driven activation) to guide meaningful behaviors. Second is the system of semantic control, which manipulates the activation of stored representations in a goal-oriented and task-dependent manner. Consequently, the semantic control system has been proposed to exert executive semantic processing that aids or constrains propagating semantic activation, enabling cognitive flexibility^2,4–6^.

Research using neuroimaging and evidence from brain-injured patients imply that semantic representation and controlled semantic processing are supported by distinct cortical networks in temporal-parietal and prefrontal areas, respectively^2,3,7,8^. Nevertheless, semantic cognition emerges form an interaction of these two neurocognitive systems, requiring a well-tuned functional coupling between them. Large-scale oscillatory phenomena have been considered a biologically plausible mechanism governing functionally organized brain processes related to complex behaviors and cognition^9–12^. Indeed, it has become increasingly evident that synchronous neural oscillations in specific frequency bands are coupled with distinct cognitive functions^13,14^. Recently, their causal role has been supported by studies using non-invasive brain stimulation^15^, indicating that neural oscillations are not an epiphenomenon of neural activity but an integral neurobiological mechanism by which the brain implements cognitive functions.

With respect to lexical–semantic processes, the most prominent oscillatory changes have been found in the theta frequency range (4–8 Hz). Implementing large-scale functional integration^16^, theta synchronization is believed to support the communication among distant cortical and subcortical regions which are involved in language^7,17^. In particular, changes in the theta-band synchronization have been repeatedly observed during lexical–semantic retrieval, indicating that theta could mediate the activation, propagation, and coupling of lexical–semantic representations distributed over the cortex^18–20^, which has been attributed to semantic representation system.

On the other hand, cortical theta synchronization has also been consistently associated with cognitive control processes. It is thought to be a mechanism by which the high-order cognitive systems exert top-down modulation of broad brain networks, especially in situations involving increased processing demands, novelty, interference control, or error monitoring^21^. Correspondingly, an increased theta synchronization has been coupled with higher processing demands during lexical–semantic retrieval (e.g., retrieving weakly-related or unrelated words, or maintaining an extended sentence context, see refs. 22 and 23). These findings indicate that theta synchronization may be generally associated with an involvement of domain-general executive control or controlled semantic processing rather than lexical-semantic representation.

Thus, although theta oscillations are clearly associated with lexical–semantic retrieval, their functional role remains poorly understood. We approached this issue using transcranial alternating current stimulation (tACS), which has been shown to effectively modulate endogenous brain oscillations and coupling of functionally related brain networks^24–28^. A tACS at 6 Hz frequency (θ-tACS) was delivered between left dorsolateral prefrontal cortex and left temporal-parietal cortex, in order to entrain theta oscillations in the prefrontal and perisylvian structures involved in controlled and automatic lexical–semantic processes^7,29^. Importantly, it has been shown that some cognitive effects of tACS depend on the relative phase of the applied currents, which may critically influence interactions among the stimulated brain networks^26,30–32^. Drawing upon this evidence, we manipulated the phase of the stimulation applied to the prefrontal and temporal-parietal regions in order to reduce or enhance long-range theta phase synchrony by an *anti-phase* θ-tACS (phase lag = 180°) or an *in-phase* θ-tACS (phase lag = 0°), respectively. Our expectation was that the phase of tACS (i.e., 180° vs. 0° lag) would interact with semantic retrieval conditions (i.e., associative vs. dissociative). The lexical-semantic retrieval was assessed using the associative chain test^33^ (ACT) and verbal fluency measures. The ACT implements a novel word-production approach by which automatic–associative and controlled–dissociative retrieval processes can be assessed and disentangled, which corresponds to the two principal neurocognitive systems proposed to constitute semantic cognition in the current model^2,5,8^. Moreover, using a response time subtraction method, additional measures reflecting net estimates of controlled semantic functions, such as response inhibition and switching, can be derived. If theta band synchronization subserves the activation or coupling within the semantic system, we expected that associative performance would be disrupted by the *anti-phase* θ-tACS (i.e., decreased long-range theta synchrony of the prefrontal and temporal-parietal cortices). However, we expected that it would be improved with *in-phase* θ-tACS (i.e., increased long-range theta synchrony) and the reversed effects would be found on the dissociative measures. On the other hand, if theta oscillations have a role in controlled semantic processing, we expected that dissociative measures would be predominantly affected, showing an improvement with the *in-phase*, but a disruption with the *anti-phase* stimulation.

## Methods

### Participants

Eighteen participants (9 males; age 22.3 ± 2.2 years) completed three experimental sessions and received three tACS conditions (*sham*, *anti-phase*, *in-phase*) in a counterbalanced order. The sessions were separated from each other by at least 6 days (mean wash-out period 7.3 ± 1.1 days). All participants were right-handed (Edinburgh Handedness Inventory – short form^34^, mean score 91.7 ± 10.4), with no history of a neurologic disease, psychiatric conditions, or current use of medication. Research has been conducted in accordance with the Declaration of Helsinki and was approved by the Ethics Committee at the Institute of Normal and Pathological Physiology, Slovak Academy of Sciences. Written informed consent was obtained from all participants. All methods were carried out in accordance with the relevant guidelines and regulations. Financial compensation was provided for participating in the study.

### Transcranial alternating current stimulation

**Figure 1:**
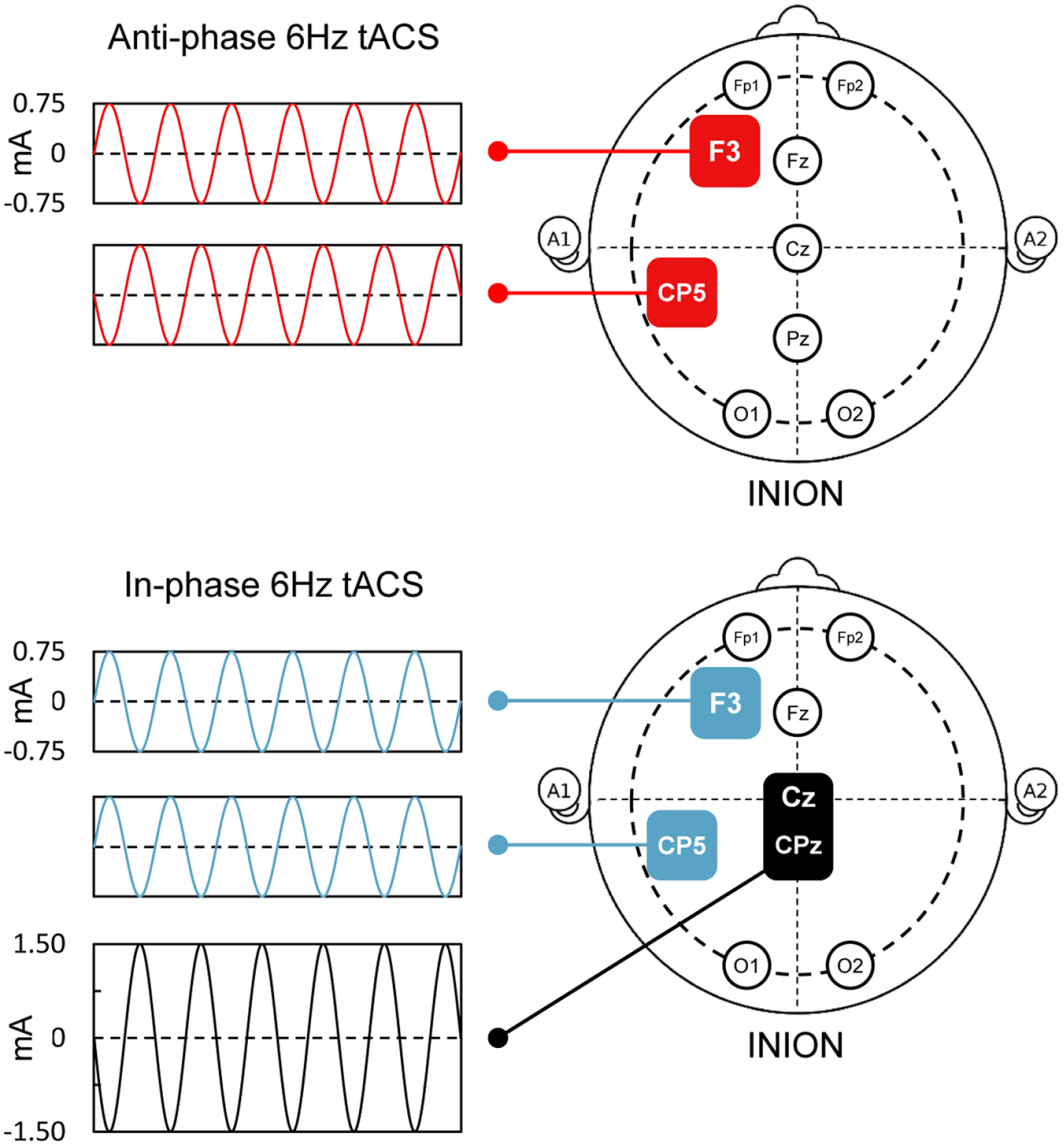
tACS electrode placement. In the *anti-phase* condition, a 6 Hz frequency stimulation was delivered using two 5 × 5 cm^2^ electrodes over F3 and CP5 with a 180° relative phase. In the *in-phase* condition, a 6 Hz stimulation was delivered using two 5 × 5 cm^2^ electrodes over F3 and CP5 with a 0° relative phase and one 7 × 5 cm^2^ return electrode aligned between the Cz–CPz location. Electric field models for both tACS conditions are shown in Supplementary Fig. S2 (see Supplementary Information).

Stimulation was delivered using a certified battery-driven current stimulator (DC-STIMULATOR PLUS, NeuroConn, Illmenau, Germany) and conductive rubber electrodes which were attached under an EEG cap using a conductive electrode paste (Ten20, Weaver and Co., Aurora, CO, USA). Three different tACS conditions were applied for each participant across three experimental sessions in a pseudo-randomized cross-over design (3 × 3 orthogonal Latin square; see Supplementary Information, Supplementary Tables S1–S3). In the *anti-phase* condition, a sinusoidal stimulation at 6 Hz with no DC offset and an intensity of 1.5 mA (peak to peak) was applied via two 5 × 5 cm^2^ electrodes (yielding peak current density of 0.03 mA/cm^2^) located over F3 and CP5 of the international 10–10 system of EEG electrode placement. The phase difference between F3 and CP5 was 180° (see Fig. 1). The *in-phase* condition was achieved by splitting one electrode cable into two channels with 0° relative phase and equal impedance (see Supplementary Information, Supplementary Fig. S1). The stimulation was applied using two 5 × 5 cm^2^ electrodes placed over F3 and CP5 and one 5 × 7 cm^2^ reference electrode centered between Cz and CPz (see Fig. 1). The peak current density under the F3 and CP5 electrodes was matched with the *anti-phase* condition (i.e., 0.03 mA/cm^2^, 1.5 mA); peak current density under the reference electrode was 0.043 mA/cm^2^. The average impedance during the stimulations was 6.4 ± 3.5 kΩ. Forward models of the peak electric field distributions for both tACS conditions were computed using SimNIBS software^35^ and are shown in Supplementary Fig. S2 (see Supplementary Information). Notably, the use of *anti-*phase and *in*-phase electrode setup has been shown effective in a number of previous research^26,30–32,36^. The duration of both active stimulation conditions was 15 min with a fade in/out period of 10 s. The *sham* stimulation had the same electrode setup as the active conditions (equal probability of either placement) but the stimulation was applied only for first 30 s. After stimulation offset, in each session participants rated perceived stimulation intensity using a Likert scale ranging from 0 (“low intensity”) to 10 (“high intensity”). There were no significant differences in the mean intensity ratings among the three tACS conditions [mean score 2.39 ± 2.12, 3.17 ± 2.55, and 4.17 ± 2.48 for *sham*, *anti-phase*, and *in-phase*, respectively; assessed using a LMEM, *F*(2,51) = 2.17, *p* = 0.125], indicating that the blinding was effective. Also, the intensity scores were not associated with the dependent measures from ACT [evaluated using a LMEM, *F*(1,5385) = 0.87, *p* = 0.351]. Additionally, adverse effects were assessed immediately after the intensity rating (four Likert scales: itching, burning, tingling, and pain; ranging from 0 – “not at all” to 10 – “very much”). Overall, the stimulation was well tolerated (mean ratings 0.40 ± 0.33, 0.53 ± 0.52, and 0.83 ± 0.51, for *sham*, *anti-phase*, *in-phase*, respectively) and none of the participants reported disruptive discomfort during or after the stimulation. However, the mean ratings were statistically different [assessed using a LMEM, *F*(2,51) = 4.09, *p* = 0.022]. Tukey corrected post-hoc tests (two-sided) revealed that participants in the *in-phase* condition reported more intensive adverse effects than in the *sham* condition, *t*(51) = 2.78, *p* = 0.020 (the other two comparisons were not significant, *p* > 0.13). Importantly, the adverse scores were not associated with the retrieval measures [evaluated using a LMEM, *F*(1,5386) = 0.40, *p* = 0.526].

### Lexical–semantic processing

**Figure 2:**
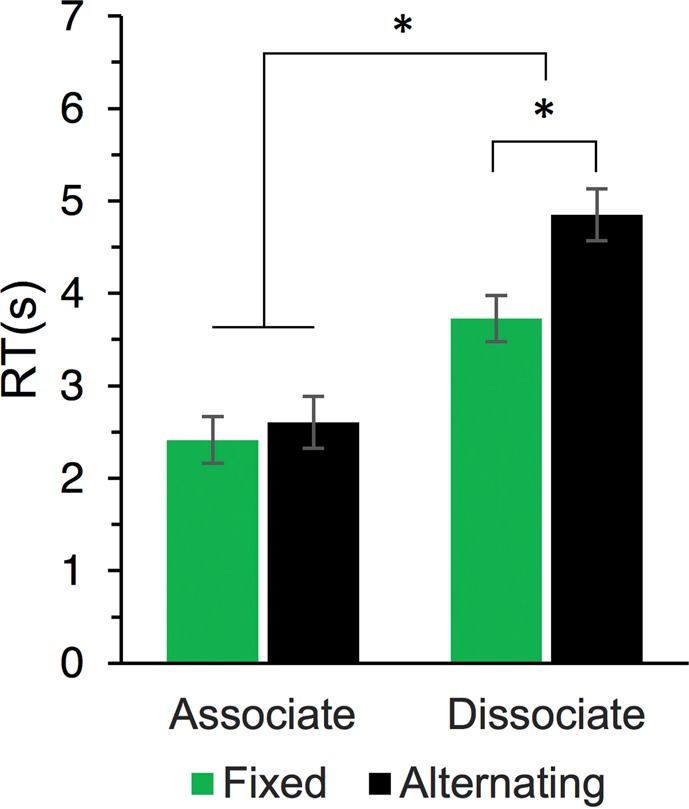
Mean RT (±1*SE*) in the ACT as a function of Response type (associate vs. dissociate) and Sequence type (fixed vs. alternating). Only the data from *sham* tACS were included. *Tukey adjusted post-hoc *p* < 0.05 (two-sided). An alternative descriptive depiction of the data is provided in Supplementary Fig. S3 (see Supplementary Information).

Lexical–semantic processing was assessed using the associative chain test^33^ (ACT). Participants continuously generated word chains according to specific rules. In the *associate chain*, participants produced words so that each new response was semantically related to the previous one (e.g., *“Hospital – Doctor – Health – Sport…”*) for 90 s. Participants were instructed that producing an unrelated word would be considered as an error. In the *dissociate chain*, participants were told to generate words so that each new response was not related to the previous one (e.g., *“Teacher – Kitchen – Hockey – Apple…”*) for 120 s. Participants were instructed that delivering a related word would count as an error. In the *associate–dissociate* chain, participants were asked to deliver associations and dissociations in alternation (i.e., switching between the two previously described rules after each response; e.g., *“Phone – Call – Banana – Monkey…”*) for 180 s. With respect to word production, the ACT thus includes two independent variables (factors) with two levels each: the *response type* (associative or dissociative) and the *sequence type* (fixed or alternating). In addition, we assessed category retrieval, requiring participants to name as many words belonging to a certain category as possible within 120 s. The category retrieval was introduced as a separate rule at the beginning of the ACT. In all retrieval conditions, participants were instructed to maintain fluent word production, not to repeat the same words within the same chain and to ignore grammatical or typing errors. Each chain started with the presentation of a word/category from a list (three word lists, referred to as ACT blocks, were created and balanced across the sessions and tACS conditions; see Supplementary Information, Supplementary Table S4). Then, each generated word was assessed for response time (RT; time required to initiate word responses using a computer keyboard). Furthermore, three additional measures were derived: (i) *response initiation* RT was calculated across all conditions that required generating related words (i.e., category retrieval RT, associate fixed RT, and associate alternating RT); (ii) *inhibition cost* was assessed as the average RT difference between delivering dissociative versus associative responses (i.e., inhibition cost = dissociative RT – associative RT), separately for the fixed and the alternating sequence type; and (iii) *switching cost* was calculated as RT difference between alternating versus fixed sequences (i.e., switching cost = alternating RT – fixed RT) in the dissociate chains only (for associate chains, there was no significant switching cost; see Fig. 2). A short exercise for each rule was provided approximately 2 min after the stimulation onset. The assessment of lexical*–*semantic measures started after 5 min of the stimulation and finished before the ramping-down period.

### Control cognitive tests

Prior to tACS, baseline short term memory and verbal fluency performance was assessed using the letter span task (LST) and the phonemic fluency test (PFT). LST required participants to remember a set of random consonants presented in 0.5 s intervals on a computer screen and recall them in the same order after a 1 s delay (the responses were provided using a keyboard). The initial span (3 consonants) changed adaptively according to the partcipant’s performance during the task: after each correct recall, the span increased by 1, whereas incorrect recalls decreased the span by 2 (the minimum was 2). This procedure continued until participants made 5 errors. Then, short term memory capacity was estimated as the average span of the 5 incorrect recalls −1. A short training session was provided at the beginning of the task. Intraclass correlation coefficient indicated high test-retest reliability of LST (*ICC* = 0.94). A repeated measures ANOVA showed that participants’ baseline short term memory capacity was not significantly different among the three tACS conditions, *F*(2,34) = 0.23, *p* = 0.798. In PFT, participants were required to write in as many nouns starting with a letter as possible within 60 s using a computer keyboard. Each PFT block included three letters, which were balanced across the sessions (letters D, O, and U for block A; letters L, F, and I, for block B; and letters K, T, A for block C). Proper nouns were considered an error. A short training session was provided at the beginning of the task. Intraclass correlation coefficient indicated high test-retest reliability of PFT (*ICC* = 0.81). A repeated measures ANOVA showed that participants’ baseline verbal fluency was not significantly different among the three tACS conditions, *F*(2,34) = 0.45, *p* = 0.633 (corrected for the PFT block factor).

### Control affective ratings

Participants’ emotional state was assessed in each session before the stimulation. Participants were asked to rate their current affect using 16 Likert scales (range from 0 – “Not at all” to 6 – “Very much”), that indicated *distress* (“tension”, “stress”, “restlessness”, and “worries”; *ICC* = 0.904), *vigilance* (“arousal”, “alertness”, “energy”, and “concentration”; *ICC* = 0.818), *task engagement* (“enthusiasm”, “motivation”, “curiosity”, and “dedication”; *ICC* = 0.851), and *self-confidence* (“confidence”, “strength”, “courage”, and “competence”; *ICC* = 0.791). A repeated measures ANOVA showed that there were no statistically significant differences in the ratings among the tACS conditions, *F*(2,34) < 1.46, *p* > 0.201.

### Data processing and analysis

The data were processed in R studio (RStudio Team, 2018) using R language and R environment (R Core Team, 2018). Prior to statistical analyses, ACT responses with large RTs (>20 s) were removed (less than 0.05%). The remaining responses were then evaluated by three independent raters for accuracy (i.e., identifying responses that did not belong to the respective category, unrelated words in associative conditions, and related words in dissociative conditions). For each response, the raters indicated whether it was valid or invalid. Any response marked invalid by more than 1 rater was removed from further analyses (less than 5% of responses were removed this way; inter-rater agreement was acceptable, *ICC* > 0.80). Due to high overall accuracy of responses (i.e., > 95%), only analyses on RT were performed. The retained RT values were winsorized (10% quantile two-sided trimming) separately for each individual, ACT block, and tACS condition. Thereafter, we evaluated the effects of tACS on ACT measures using linear mixed-effect models (LMEM). Assuming that the specific production rules in ACT engage different neurocognitive processes, a separate LMEM was computed for each ACT condition (referred to as the “basic” measures). The statistical significance was assessed using χ^2^ likelihood ratio tests at 0.05 level. Furthermore, response initiation, inhibition cost, and switching cost scores (referred to as the “derived” measures) were derived from the basic measures and evaluated using LMEMs. The family-wise error rate due to multiple statistical tests (*N* = 8) was controlled for by applying sequential Bonferroni corrections (adjusted *p*-values are reported). Post-hoc pairwise comparisons between the tACS conditions were evaluated using Wald’s statistic and Satterthwaite approximation of degrees of freedom. The resulting *p*-values were corrected using Tukey HSD adjustment (two-sided tests). Effect sizes of the within-subject stimulation effects were estimated using Cohen’s *d*_RM_ (see Supplementary Information for more details).

## Results

### Effects of the ACT factors on the retrieval RT

First, we investigated the effects of Response type (associative vs dissociative) and Sequence type (fixed vs alternating) on RT in the ACT, using only the data from *sham* condition (i.e., not affected by tACS stimulation). A LMEM with a random intercept for participants showed significant main effects of Response type, χ^2^(1) = 439.0, *p* < 0.001, Sequence type, χ^2^(1) = 62.4, *p* < 0.001, and their interaction, χ^2^(1) = 31.1, *p* < 0.001. As shown in Fig. 2, the dissociative RT was substantially higher than the associative RT and the alternating condition further increased the dissociative RT.

### The effects of tACS on the basic ACT measures

**Figure 3:**
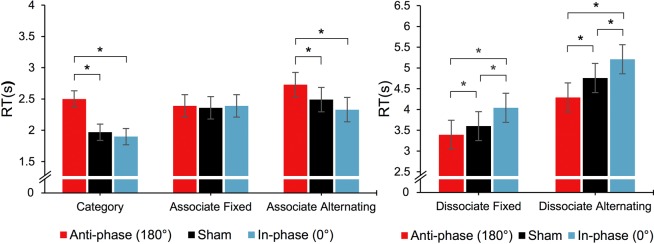
Mean RT (±1*SE*) of the ACT measures as a function of tACS condition. Left panel: associative conditions, right panel: dissociative conditions. The *anti-phase* θ-tACS (red bars) had a negative effect on category and associative alternating RT, but facilitated dissociative RT. The *in-phase* stimulation (blue bars) increased dissociative RTs. *Tukey adjusted post-hoc *p* < 0.05 (two-sided). An alternative descriptive depiction of the data is provided in Supplementary Fig. S4 (see Supplementary Information).

Next, we investigated the effect of tACS on the basic ACT measures. Using LMEMs, the RTs were modeled as a function of tACS (the fixed factor of interest), while controlling for ACT block (a control fixed factor) and random subject intercepts. The model for the category retrieval RT showed a statistically significant effect of tACS, χ^2^(2) = 38.1, *p* < 0.001. Post-hoc tests revealed that the average RT was significantly higher in the *anti-phase* than in the *sham* and the *in-phase* stimulation (*p* < 0.001 for both, Fig. 3). The RT in the *in-phase* stimulation was not statistically different from the *sham* stimulation (*p* = 0.887). The model for the associative fixed RT showed no significant effects of tACS, χ^2^(2) = 0.37, *p* = 0.832. However, tACS significantly affected the associative alternating RT, χ^2^(2) = 19.4, *p* < 0.001. Post-hoc tests revealed that the average RT was significantly higher in the *anti-phase* than in the *sham* and the *in-phase* stimulation (*p* = 0.032 and *p* < 0.001). Although the average RT was shorter in the *in-phase* than in the *sham* stimulation, this difference was not statistically significant after the post-hoc correction (*p* = 0.129). A LMEM for the dissociative fixed RT showed a significant effect of tACS, χ^2^(2) = 46.3, *p* < 0.001. Post-hoc tests revealed that the average RT was significantly shorter in the *anti-phase* than in the *sham* stimulation (*p* = 0.022), which was in turn significantly shorter than in the *in-phase* stimulation (*p* < 0.001). The difference between *anti-phase* and *in-phase* was also significant (*p* < 0.001). The same pattern was also observed for the dissociative alternating RT, χ^2^(2) = 40.6, *p* < 0.001, as indicated by the post-hoc tests (*p* < 0.001 and *p* = 0.013, respectively). See Fig. 3 and Table 1 for more details.

**Table 1:** Summary of the θ-tACS effects on the lexical–semantic measures.

Measure			Estimated mean (SE)^a^			Test^b^		Effect size^c^	
			*Sham*	*Anti-phase*	*In-phase*	χ^2^	*p*	180°-Sham	0°-Sham
Basic	Category retrieval		2.01 (0.13)	2.47 (0.13)	1.97 (0.13)	38.1	<0.001	0.63*	0.04
	Associate	Fixed	2.38 (0.18)	2.40 (0.18)	2.44 (0.18)	0.37	0.832	0.18	0.06
		Alternating	2.49 (0.19)	2.70 (0.19)	2.33 (0.19)	19.4	<0.001	0.37*	0.36
	Dissociate	Fixed	3.96 (0.36)	3.71 (0.36)	4.38 (0.36)	46.3	<0.001	0.62*	0.72*
		Alternating	4.98 (0.39)	4.39 (0.39)	5.40 (0.39)	40.6	<0.001	0.47*	0.73*
Derived	Response initiation		2.23 (0.22)	2.59 (0.22)	2.21 (0.22)	9.21	0.030	0.55*	0.02
	Inhibition cost		2.04 (0.42)	1.34 (0.42)	2.68 (0.42)	20.0	<0.001	0.77*	0.52
	Switching cost		1.10 (0.25)	0.67 (0.25)	1.02 (0.25)	2.39	0.303	0.07	0.36

^a^RT means (±1*SE*) of the respective LMEM as a function of tACS; ^**b**^Likelihood-ratio based *p-*value adjusted by sequential Bonferroni correction; ^c^Cohen’s *d*_*RM*_ for the *anti-phase* versus *sham* (180°-Sham) and the *in-phase* versus *sham* (180°-Sham) contrasts. *Post-hoc Tukey corrected *p* < 0.05 (two-sided).

### The moderating role of difficulty in category retrieval

**Figure 4:**
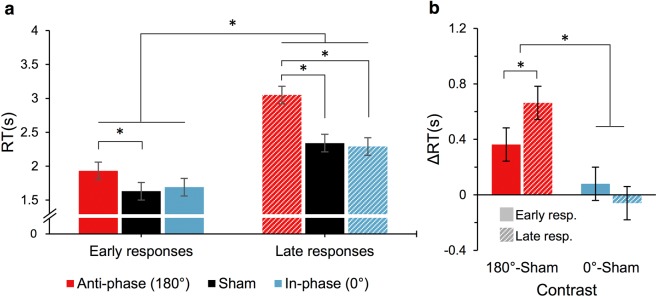
The effects of θ-tACS on the category retrieval for early and late responses (**a**). The *anti-phase* versus *sham* (180°–*sham*, red) and the *in-phase* versus *sham* stimulation (0°–*sham*, blue) contrasts of the effects as a function of the response serial position (**b**). The negative effect of the *anti-phase* stimulation was stronger for the later responses (stripped bars), which are usually less common for the given category. Error bars represent ± 1*SE*. *Tukey adjusted post-hoc *p* < 0.05 (two-sided). An alternative descriptive depiction of the data is provided in Supplementary Fig. S5 (see Supplementary Information).

The responses from each category chain (separately for each participant) were divided into two halves by their serial position, assuming the early responses involved more dominant and typical category associates than the later responses^37^. Subsequently, we included this serial position split in a LMEM as a factor indicating the relative difficulty of retrieval (i.e., retrieving prepotent versus less accessible category exemplars, respectively). The model confirmed that the average RT for the late category responses was substantially longer (mean 2.52 ± 0.14) than for the early responses (mean 1.79 ± 0.14), χ^2^(1) = 106.2, *p* < 0.001. Importantly, the model revealed a significant interaction of tACS and the serial position, χ^2^(2) = 8.51, *p* = 0.014, indicating that the negative effect of the *anti-phase* tACS was stronger for the late category responses (see Fig. 4).

### The effects of tACS on the derived ACT measures

**Figure 5:**
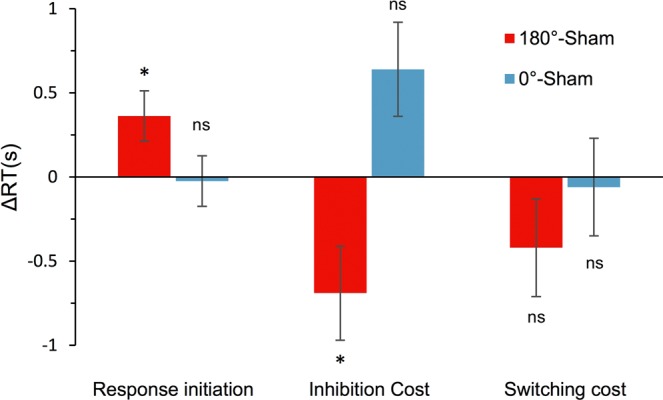
The *anti-phase* versus *sham* (180°–*sham*, red) and the *in-phase* versus *sham* θ-tACS (0°– s*ham*, blue) contrasts for the derived ACT measures: response initiation (left panel), inhibition cost (middle panel), and switching cost (right panel). The *anti-phase* stimulation increased response initiation RT, but decreased inhibition cost ΔRT. The *in-phase* stimulation had an opposing effect on inhibition cost ΔRT. Switching cost in dissociative responses was not significantly affected by tACS. Error bars represent ± 1*SE*. *Tukey adjusted post-hoc *p* < 0.05 (two-sided); ns – statistically non-significant effect (p > 0.05). An alternative descriptive depiction of the data is provided in Supplementary Fig. S6 (see Supplementary Information).

Finally, we investigated the effect of tACS on response initiation, inhibition cost, and switching cost (Fig. 5 and Table 1). The LMEMs included an additional random intercept for ACT block by condition term where appropriate (for more details see Supplementary Information). The model for response initiation showed a statistically significant effect of tACS, χ^2^(2) = 9.21, *p* = 0.030. Post-hoc tests revealed that the average RT was significantly longer in the *anti-phase* than in the *sham* and also the *in-phase* stimulation (*p* = 0.028 and *p* = 0.021, respectively). The model for inhibition cost showed a statistically significant effect of tACS, χ^2^(2) = 20.05, *p* < 0.001. Post-hoc tests revealed that the average RT was significantly shorter in the *anti-phase* than in the *sham* stimulation (*p* = 0.042), which was in turn shorter than in the *in-phase* stimulation, however, this did not reach statistical significance after the post-hoc correction (*p* = 0.066). The difference between the average RT in the *anti-phase* and the *in-phase* stimulation was significant (*p* < 0.001). The model for switching cost did not show a significant effect of tACS, χ^2^(2) = 2.39, *p* = 0.303.

## Discussion

Neural oscillatory activity seem to play a fundamental role in cognitive processes. The present study addressed the role of theta oscillations in lexical–semantic processing by means of non-invasive transcranial alternating current stimulation (tACS). We show that exogenous theta oscillations, applied using tACS at 6 Hz (θ-tACS) over prefrontal and posterior perisylvian cortex, substantially affect semantic retrieval performance. Moreover, we revealed that the effects of tACS were critically dependent on the retrieval rule as well as on the phase alignment of the stimulation currents between the stimulated sites. An automatic associative retrieval was inhibited by θ-tACS applied in *anti-phase*. On the other hand, a controlled dissociative retrieval was inhibited by the *in-phase* but facilitated by the *anti-phase* stimulation.

According to current neurocognitive models of semantic cognition^2,7^, initiating semantically related responses engages a relatively automatic, stimulus-driven processing, i.e., semantic activation and spreading. Dissociative production, on the other hand, requires the suppression of such pre-potent associates, resolution of stimulus-driven interference, as well as a shift towards unrelated semantic clusters, which counteract habitual responding^2,4,38^. The diverging effects of θ-tACS on the associative and dissociative measures are in line with these and also other studies, which reported differences in the cognitive and neural mechanisms of associative vs. dissociative retrieval^39,40^. In addition, our results suggest that the associative and dissociative processes are functionally antagonistic and therefore differentially affected by entraining theta oscillations.

Synchronized theta activity is a candidate neural mechanism for binding lexical–semantic representations, which rely on widely distributed neural assemblies in the cerebral cortex^2,18,20^. Furthermore, current models imply that semantic processing depends on long-range interactions between anterior (mainly “executive”) and posterior (mainly “representative”) cortical regions^1,7,41^ and theta synchronization could be a neural mechanism which mediates these interactions^23^. Our current findings are in agreement with these proposals. From this viewpoint, a plausible interpretation is that global phase alignment of theta oscillations, reinforced by the *in-phase* θ-tACS, strengthens the stability of default pre-potent semantic connections. This results in higher demands in dissociative tasks that are required to disengage from the prepotent semantically related representations (see Fig. 5). Conversely, an anti-phasic entrainment (i.e., *anti-phase* θ-tACS) results in a decreased binding of semantic networks and attenuates pre-potent semantic activations. Decreased semantic activation, in turn, alleviates the cognitive demands to overcome automatic stimulus-driven activations and thus facilitates retrieval of semantically unrelated items (see Fig. 5). Moreover, the fact that the *anti-phase* θ-tACS was more disruptive for late compared with early responses in the category retrieval task supports this account. Being less dominant, later responses induce weaker semantic activation^37,42,43^, which may be more easily disrupted and may not reach the threshold for conscious access when the neural transmission is downregulated (as during the *anti-phase* θ-tACS). On the other hand, early dominant associates provide rapid and strong semantic activation, which remains sufficiently strong even when the functional coupling of prefrontal and posterior language networks is decreased (see Fig. 4). Taken together, our findings indicate that theta oscillations may modulate the gain of semantic activation and so affect the accessibility of lexical–semantic representations during retrieval.

Alternatively, θ-tACS could also modulate post-retrieval processes pertaining to the transient maintenance (and processing) of the already accessed lexical–semantic representations in a short-term buffer. Research indicates that working memory maintenance is accompanied with synchronized theta activity in the anterior and posterior brain regions^44,45^. Interestingly, manipulating theta phase-alignment between these brain regions using tACS can facilitate or disrupt the maintenance of verbal and visual representations in working memory^26,30,46^. Results of these studies imply that the *in-phase* θ-tACS could support the stability of lexical–semantic representations in working memory and thus hinder the dissociative performance, requiring increased inhibitory demands to dissociate from the currently maintained semantic set. Such a mechanism would fit, for instance, with the proposed role of theta synchrony in the maintenance of coherent percepts^11^. On the other hand, disrupting the transiently maintained lexical–semantic set by *anti-phase* stimulation would facilitate dissociative performance (due to lower inhibitory demands) at the cost of a restricted potency of working memory content to prime semantic associates^47^.

Our results suggest that left-hemispheric fronto-temporoparietal theta synchrony does not play an important role in executive control of retrieval. If this were the case, the *in-phase* θ-tACS should have facilitated dissociative performance, whereas, the *anti-*phase stimulation should have impaired it. Our results clearly suggest the opposite. Nevertheless, this does not rule out the role of theta oscillations in cognitive control. For instance, we may speculate that a decoupling of prefrontal cortex from cortical networks (established by the *anti-phase* stimulation) could in a reciprocal manner support the interactions within frontostriatal circuits engaged in cognitive control^42^.

With respect to controlled retrieval, it should be taken into account that habitually retrieved associates during dissociative performance may evoke cognitive interference and response conflict, which has been linked with theta oscillatory activity^21^. Thus, the effects of θ-tACS on dissociative performance could potentially reflect the modulation of conflict processing (i.e., conflict monitoring, detection, and/or the implementation of a behavioral change. This, however, seems improbable since (i) it does not explain the observed effects of tACS on associative retrieval, and, (ii) the sources of conflict-related theta activity have been identified to lie primarily in the medial frontal cortex^21^, while our stimulation was directed to the lateral cortex. Nevertheless, since our behavioral task did not target semantic (in)congruency further research is needed to address this issue more specifically.

Yet, we note that as in the previous studies^26,30–32,36^, the phase-specific effects of theta entrainment should be interpreted with caution, since the electric field distributions for the tACS conditions were not fully identical (see Supplementary Fig. S2 in Supplementary Information). This was a consequence of using a common reference electrode in the *in-phase* condition, which in our case resulted in a distinct polarization near the central cortical regions. Notably, however, the study by Violante *et al*.^26^ indicates that tACS exerts effects predominantly on the active brain regions, i.e. on the neural tissue which is actively engaged by the cognitive task being performed. Given the fact that in our study the electrical field distribution was very similar in both stimulation conditions, we have a strong reason to assume that both the *anti-* and *in-phase* stimulation targeted the brain’s semantic system, in particular the left prefrontal and perisylvian regions strongly employed in semantic retrieval^2,7,29,48^. Moreover, since the semantic processing is predominantly supported by the left lateral prefrontal and temporal-parietal regions, the polarization near the central reference electrode in the *in-phase* condition is not expected to considerably influence the retrieval performance^49^. Yet, further neuroimaging and electrophysiological research is needed to resolve the current uncertainties (see Supplementary Information for extended discussion on this topic).

Finally, it is important to note that theta activity is unlikely to be the sole oscillatory brain mechanism regulating semantic cognition. Electrophysiological studies have shown that semantic processing is associated with changes across multiple oscillatory frequencies and cortical regions, suggesting that different frequency bands and their interactions might reflect (and possibly implement) distinct aspects of the retrieval function, such as memory reinstatement^50^, semantic conflict^51^, or sentence processing^22^. In particular, alpha-band (10 Hz) oscillatory activity has been associated with inhibitory functions and controlled access to stored knowledge representations^52^. Relevant for the concepts of dissociative retrieval and switching, right-hemispheric alpha oscillations have been recently considered to play a role in suppressing habitual lexical-semantic associates that facilitates flexible thinking^53^. Furthermore, beta-band (15–30 Hz) as well as gamma-band (>30 Hz) oscillations have been implicated to support semantic representation and binding of modality-specific features^20,23,54^. Therefore, the fact that the current study did not include a control tACS at the frequencies outside of the theta-range is a limitation that awaits further study. Similarly, based on the current findings, it is not possible to evaluate whether the observed effects of theta-modulation are specific to the left prefrontal and temporal-parietal network, as control montages targeting other cortical regions (e.g., right-hemispheric counterpart of this network, or frontal midline regions) were not investigated. Further research could also benefit from combining high-density electro- or magnetoencephalography with a more focused neurostimulation protocols to approach and extend the findings resulting from the current study.

## Conclusions

Semantic retrieval has been previously associated with increased theta synchronization. Here, we demonstrated that anti-phasic θ-tACS of the prefrontal and temporal-parietal cortex of the left hemisphere disrupted retrieving semantically related representations and alleviated the inhibitory demands when dissociating from habitual responses. Conversely, exogenous currents in synchronous phase-alignment hindered participants’ ability to inhibit commonly evoked associates (i.e., automatic, stimulus-driven signals), as demonstrated by increased inhibitory demands during dissociative performance. Such effects suggest that theta phase-alignment may regulate the gain of stimulus-driven semantic activation. The alternative hypothesis that the induction of synchronous theta oscillations would improve executive control of retrieval was not supported. Taken together, our findings indicate that long-distance left-hemispheric theta synchronization may constitute a neurocognitive mechanism for activation (binding) and/or maintenance of lexical–semantic representations rather than executive control of semantic retrieval.

## Supplementary information

Supplementary Information

Dataset 1

## Data Availability

The dataset analyzed during the current study is included in Supplementary Information files and also available from the corresponding author on reasonable request.
