# Supplementary material for: Neural theta oscillations support semantic memory retrieval
Source: Sci Rep. 2019 Nov 27;9:17667. doi: 10.1038/s41598-019-53813-y (PMC6881370; doi:10.1038/s41598-019-53813-y)
Supplement: Supplementary file 1 — Supplementary Information [file 41598_2019_53813_MOESM1_ESM.pdf]

# SUPPLEMENTARY INFORMATION

**Neural theta oscillations support semantic memory retrieval**

Martin Marko, Barbora Cimrová, Igor Riečanský

## Contents

1. Supplementary methods....2

2. Supplementary discussion....5

3. Supplementary figures....7

4. References....12

# 1. Supplementary methods

*1.1. Orthogonalization of the experimental conditions.*

The participants were pseudo-randomly assigned into experimental condition tACS (sham, anti-phase, in-phase) and association chain test (ACT) block (A, B, C), which were properly orthogonalized across the three experimental sessions (see Table S1 and S2, respectively) and each other (see Table S4). Word stimuli in ACT blocks are shown in Table S4.

**Table S1.** Orthogonalization of tACS conditions across experimental sessions.

| **Factors** | ***Group 1*** | ***Group 2*** | ***Group 3*** |
|---|---|---|---|
| Session 1 | 0° | 180° | Sham |
| Session 2 | 180° | Sham | 0° |
| Session 3 | Sham | 0° | 180° |

*Note:* 0° - anti-phase, 180° - in-phase.

**Table S2.** Orthogonalization of ACT Blocks across experimental sessions

| **Factors** | ***Group 1*** | ***Group 2*** | ***Group 3*** |
|---|---|---|---|
| Session 1 | A | B | C |
| Session 2 | B | C | A |
| Session 3 | C | A | B |

**Table S3.** Orthogonalization of ACT Blocks for tACS conditions

| **Factors** | ***Group 1*** | ***Group 2*** | ***Group 3*** |
|---|---|---|---|
| Sham | A | B | C |
| In-phase | B | C | A |
| Anti-phase | C | A | B |

**Table S4.** Word stimuli for ACT rules

| **Rules** | ***A*** | ***B*** | ***C*** |
|---|---|---|---|
| Category | Animals | Electronics | Occupations |
| Associate | Theatre | Music | Movie |
| Dissociate | Forest | Lake | Mountain |
| Switching | Book | Keys | Phone |

### *1.2. Statistical analyses (Linear mixed effect models)*

Effects of the ACT conditions on the retrieval RT during sham conditions (i.e., not affected by active tACS conditions) were evaluated using LMEM as *model = RT ~ RespT + SeqT + RespT:SeqT + (1 | ID)*, where *RT* were response times, *RespT* was response type (associative or dissociate), *SeqT* was sequence type (*fixed* or *alternating*), and 1 | *ID* was random intercept for subjects. This model was evaluated using *Anova()* function *car* package. The effects of tACS on the basic ACT measures (separately for category, associative fixed, associative alternating, dissociative fixed, and dissociative alternating measure) were assessed by comparing *model0 = RT ~ Block + (1 | ID)* and *model1 = RT ~ tACS + Block + (1 | ID)*, using *anova()* function from *stats* package. The *tACS* factor included 3 conditions (sham, anti-phase, in-phase). Block factor had also 3 conditions (A, B, C), representing stimulus material, that were counterbalanced across the sessions (see Table S4). Additionally, in order to assess the moderating role of difficulty in category retrieval, we used *model = RT ~ tACS * Half + Block + (1 | ID)* formula. The *Half* factor had two conditions (first, second). The model was evaluated using *Anova()* function. The effects of tACS on response initiation were evaluated by comparing *model0 = RT ~ (1 | ID) + (1 |Block:Condition)* and *model1 = RT ~ tACS + (1 | ID) + (1 |Block:Condition)*, using *anova()* function. In this model, the *Condition* factor had two levels (category, associative) and was used to account for the differences between these two ACT conditions. The same syntax was used for inhibition cost. In this model, the *Condition* factor included two levels (fixed, alternating). Finally, switching was evaluated by comparing *model0 = RT ~ (1 | ID) + (1 | Block)* and *model1 = RT ~ tACS + (1 | ID) + (1 | Block)*, using *anova()* function. Notably, switching effect was only present in the dissociative response type and therefore the *Condition* term (associative, dissociative) was not included. The LMEM syntax is listed in see Table S5.

**Table S5.** LMEM model structure and syntax

| Effects | Syntax |
|---|---|
| Effects of ACT conditions on RT (in sham) | RT ~ **RespT** * **SeqT** + (1 \| ID) |
| Effects of tACS on RT in basic measures [a] | RT ~ **tACS** + Block + (1 \| ID) [b] |
| Effects of tACS on RT in derived measures [b] | RT ~ **tACS** + (1 \| ID) + (1 \| Block:Condition) |

[a] – the basic measures were: category, associative fixed, associative alternating, dissociative fixed, and dissociative alternating RTs; [b] – for category RT, the interaction of tACS and serial position was assessed as RT ~ tACS * Half + Block + (1 | ID). [c] – the derived measures were: response initiation, inhibition cost, and switching cost. The main factor(s) of interest are marked in bold.

*Computation of the effect size*. For the *anti-phase* versus sham contrast and *in*-phase versus sham contrast, effect sizes were calculated as $d_{RM} = M_{diff} / SD_{dif}$, where $M_{diff}$ is the sample mean change (average RTs in the respective active condition minus average RTs in the sham condition) and $SD_{diff}$ represents the sample standard deviation of change scores[1]. Notably, winsorized means and standard deviation (10% quantile two-sided trimming) were used in order to obtain robust effect size estimates[2].

*Used R packages*

- ***lme4*;** Douglas Bates, Martin Maechler, Ben Bolker, Steve Walker (2015). Fitting Linear Mixed-Effects Models Using lme4. *Journal of Statistical Software*, 67(1), 1-48.
- ***lmerTest;*** Kuznetsova A, Brockhoff PB, Christensen RHB (2017). LmerTest Package: Tests in Linear Mixed Effects Models. *Journal of Statistical Software*, 82(13), 1–26.
- ***lsmeans***; Russell V. Lenth (2016). Least-Squares Means: The R Package lsmeans. *Journal of Statistical Software*, 69(1), 1-33.
- ***multcomp***; Torsten Hothorn, Frank Bretz and Peter Westfall (2008). Simultaneous Inference in General Parametric Models. *Biometrical Journal* 50(3), 346--363.
- ***psych;*** Revelle, W. (2018) psych: Procedures for Personality and Psychological Research, Northwestern University, Evanston, Illinois, USA.

## 2. Supplementary discussion

Targeting inter-regional phase synchronization using *in-phase* and *anti-phase* montages has been a critical concern in a number of previous studies using tACS [3–8]. The main issue is that stimulating *in-phase* requires a reference electrode, which introduces an additional field polarization within the underneath and nearby cortical regions. As a consequence, the *in-phase* and *anti-phase* usually yield more or less unbalanced electric field distributions, which may confound or interact with the phase-specific tACS effects.

On the other hand, however, several lines of recent evidence concur that neurobiological and cognitive effects of tES depend on the brain activation pattern that occurs during the stimulation rather than the distribution of induced electrical fields. In fact, it has been proposed and repeatedly verified that tES preferentially modulates functional networks that are engaged by the task performed during the stimulation [10,11] (see Bikson & Rahman, 2013 for a short review). As an important example, in a recent study, Pisoni et al. (2018) have provided evidence that even though the currents delivered using tDCS spread far from the stimulation sites (as assessed by field density models), the functional effects on neuronal processing and cognition are restricted to those areas which are engaged during the stimulation. In a very similar fashion, Violante et al. (2017) demonstrated that tACS-induced brain activity and connectivity effects were restricted to task-related functional brain network rather than the precise placement of electrodes. Notably, as in the case of our study, Violante and colleagues used an extra reference electrode to achieve *in-phase* stimulation, but the cortical areas underneath and nearby this site were not affected by the stimulation (as revealed by fMRI), despite the fact that these cortical areas received the strongest field polarization (i.e., the sum of the currents from the two *in-phase* electrodes). Therefore, in line with the evidence from these studies, although the field polarization of the brain in the *in*-phase and the *anti*-phase conditions were not completely overlapping, the minor differences should not account for the cognitive effects observed in our study. (Please also note that from the abovementioned studies also imply that even a single electrode montage may produce dissimilar pattern of excitability and/or connectivity if the brain is engaged by distinct cognitive tasks).

Furthermore, as recently emphasized by Saturnino and colleagues (2017), the unwanted field polarization near the reference electrode may represent a confound only if it overlaps with the functional network that implement the dependent behavioral measure. Since semantic memory retrieval is almost exclusively supported by a left-lateralized brain network, the central scalp was selected as the most appropriate site for the reference electrode in our study in order to minimize the relevance of the additional unwanted polarization.

Finally, it has been proposed that a dual-site high-definition tACS (HD-tACS) may represent a more appropriate solution for targeting inter-regional phase synchronization [13] (note that high-definition montages usually include 4x1 electrodes, where 4 outer electrodes surround 1 central electrode that has the opposite polarity/phase; dual-site HD montages have also been used

for tDCS [14]). The main reason is that by using dual-site HD-tACS it is possible to maintain the same electric field distributions for both the *in-phase* and the *anti-phase* condition and thus unambiguously interpret the phasic manipulation. However, as acknowledged by Saturnino et al. (2017), this applies insofar the distances among the outer electrodes of the two respective HD montages are sufficiently large (crucial is the smallest distance between the two outer electrode "rings"). Otherwise, provided that the polarity of the outer-ring electrodes is opposite (i.e., *anti-phase*), the cortical areas between the two HD-tACS sites will be considerably polarized. Importantly, if the electrodes are close and have the same phase (i.e., *in-phase*), no such polarization would occur, resulting in slightly dissimilar field distributions between the conditions. Nevertheless, an optimized dual-site HD-tACS may be a preferable solution for studies targeting phase synchronization of more distant brain regions [15]. On the other hand, however, the dual-site HD-tACS solution may be less efficient for modulating white matter connections that mediate such inter-regional brain interactions. Further research is required to optimize tACS protocols to provide unambiguous manipulation of cortical phase-relations.

## 3. Supplementary figures

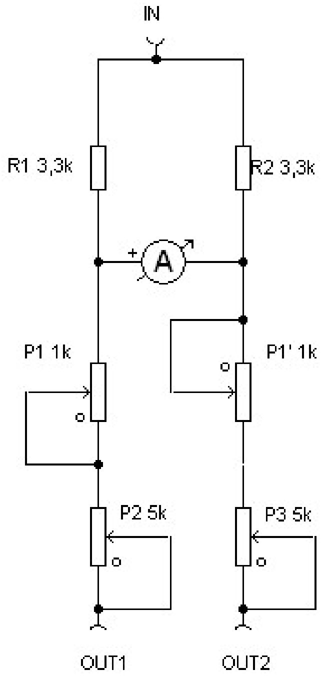


**Fig. S1.** *In-phase* cable splitter. For the *in-phase* stimulation one electrode cable was split into two channels with 0° relative phase. One fixed and two variable resistors (rheostats, one for coarse and the second for fine-grained adjustments) were serially connected with both channels. The difference in voltage between the channels was indicated by a galvanometer, which could be adjusted to ensure even current intensity between the two *in-phase* channels. Please note that the circuit includes only passive elements and thus in no way increases the safety or tolerability risks of the stimulation.

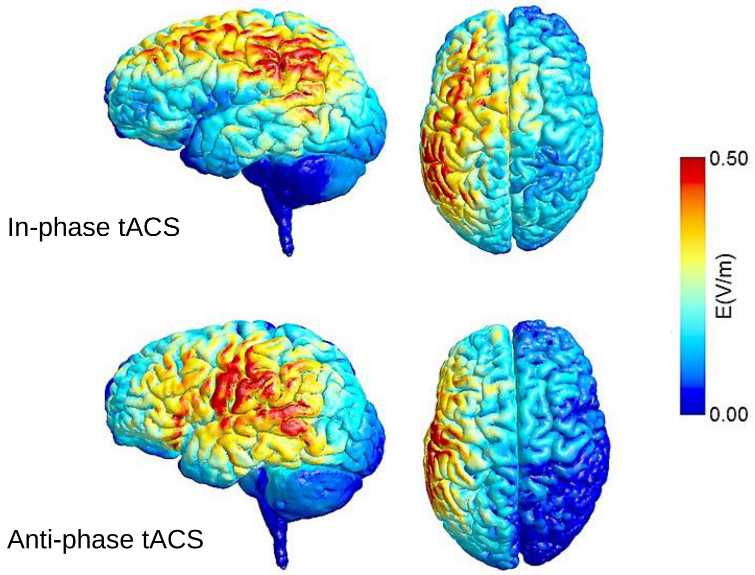


**Fig. S2.** Computational forward model of the peak electric field intensity for the *in-phase* (upper panel) and the *anti-phase* tACS (lower panel). In both conditions, two 5x5 $cm^2$ electrodes were located over F3 and CP5 of the international 10-10 system of EEG electrode placement. The *in-phase* condition included an additional 5x7 $cm^2$ reference electrode centered between Cz and CPz. The figure depicts left-lateral and dorsal view of the estimated field intensity.

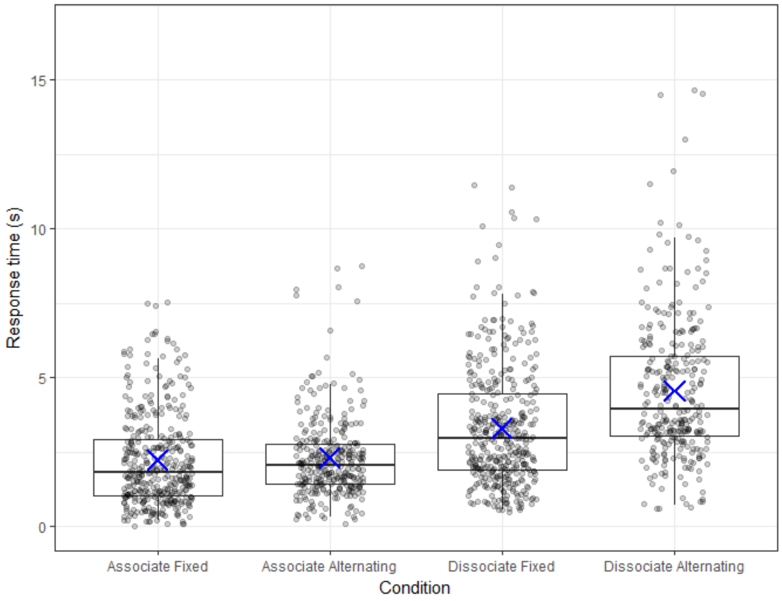


**Fig. S3.** Boxplots showing response times for the ACT conditions during sham tACS (*n* = 376, 307, 394 and 292, for the respective ACT conditions) Notes: individual RTs were jittered (parameter = 0.2); blue "X" marks indicate mean values.

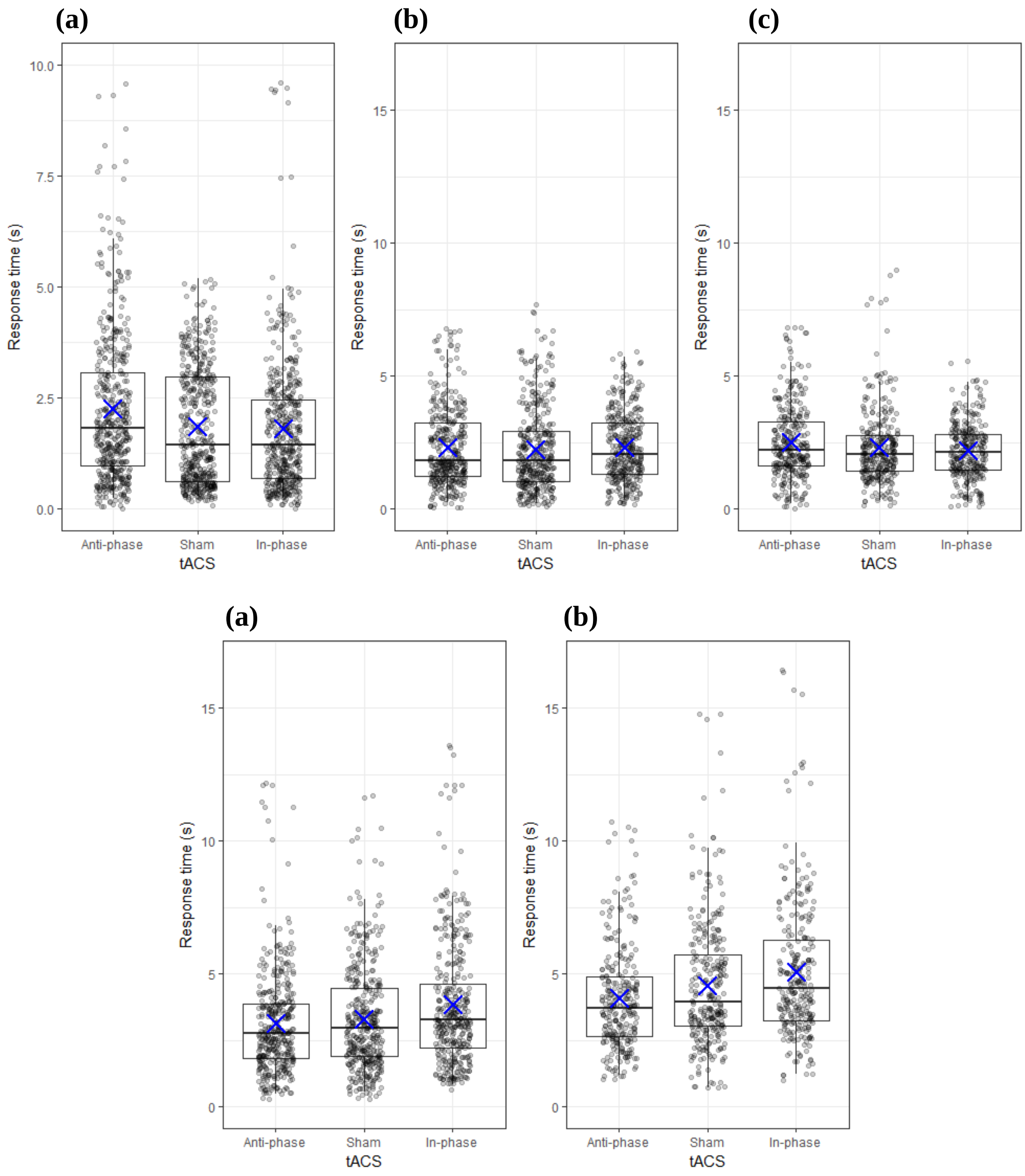


**Fig. S4.** Boxplots showing response times for the basic ACT retrieval measures as a function of tACS: (a) category (*n* = 467, 468, and 459, for the respective tACS condition), (b) associate fixed (*n* = 367, 376, and 351), (c) associate alternating (*n* = 308, 307, and 298), (d) dissociate fixed (*n* = 388, 394, and 370), and (e) dissociate alternating (*n* = 276, 292, and 271). Notes: individual RTs were jittered (parameter = 0.2); blue "X" marks indicate mean values.

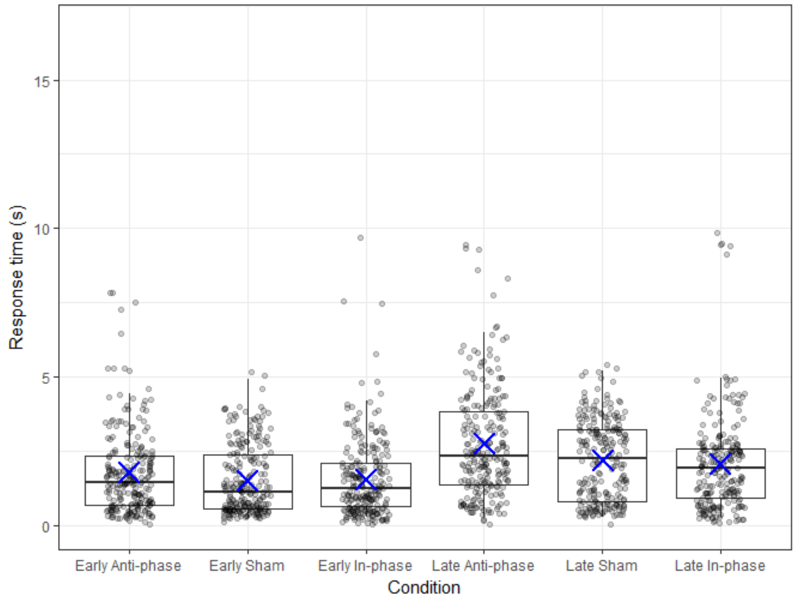


**Fig. S5**. Boxplots showing category retrieval response times for for early and late responses as a function of tACS ($n$ = 237, 239, 232, 230, 229, and 227, for the respective conditions). Notes: individual RTs were jittered (parameter = 0.2); blue "X" marks indicate mean values.

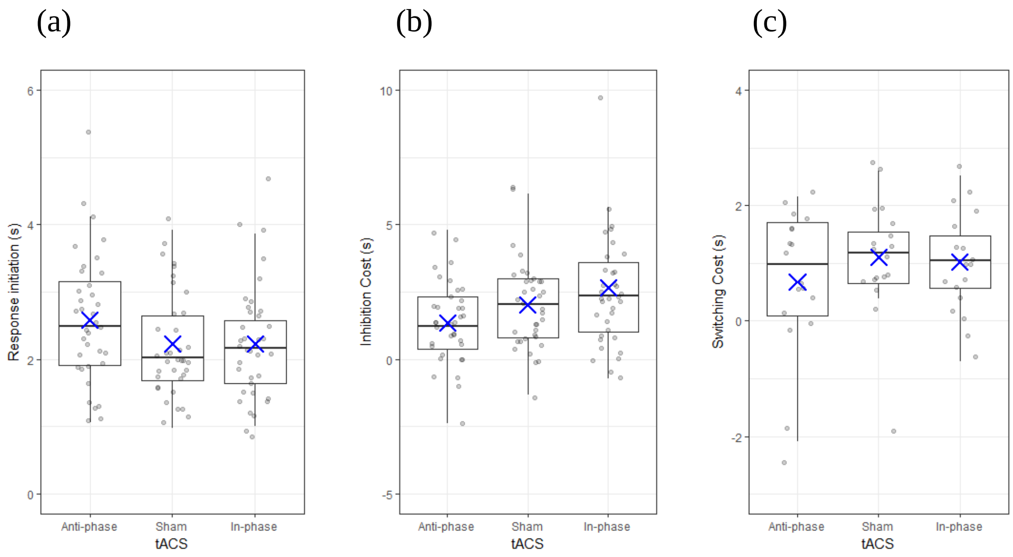


**Fig. S6.** Boxplots showing response times for the derived ACT measures as a function of tACS: (a) response initiation ($n$ = 36 for each tACS condition), (b) inhibition cost ($n$ = 36 for each tACS condition), and (c) switching cost ($n$ = 18 for each tACS condition). Notes: points indicate averaged RTs of the corresponding measure for each individual. The average RTs were jittered (parameter = 0.2); blue "X" marks indicate mean values.

## 4. References


1. Morris, S. B. & DeShon, R. P. Combining effect size estimates in meta-analysis with repeated measures and independent-groups designs. *Psychol. Methods* **7,** 105–125 (2002).
2. Algina, J. & Penfield, R. D. An Alternative to Cohen' s Standardized Mean Difference Effect Size: A Robust Parameter and Confidence Interval in the Two Independent Groups Case. *Psychol. Methods* **10,** 317–328 (2005).
3. Polanía, R., Nitsche, M. A., Korman, C., Batsikadze, G. & Paulus, W. The importance of timing in segregated theta phase-coupling for cognitive performance. *Curr. Biol.* **22,** 1314–1318 (2012).
4. Alekseichuk, I., Pabel, S. C., Antal, A. & Paulus, W. Intrahemispheric theta rhythm desynchronization impairs working memory. *Restor. Neurol. Neurosci.* **35,** 147–158 (2017).
5. Violante, I. R. *et al.* Externally induced frontoparietal synchronization modulates network dynamics and enhances working memory performance. *Elife* **6,** 1–22 (2017).
6. Tseng, P., Iu, K.-C. & Juan, C.-H. The critical role of phase difference in theta oscillation between bilateral parietal cortices for visuospatial working memory. *Sci. Rep.* **8,** 349 (2018).
7. Tseng, P., Chang, Y.-T., Chang, C.-F., Liang, W.-K. & Juan, C.-H. The critical role of phase difference in gamma oscillation within the temporoparietal network for binding visual working memory. *Sci. Rep.* **6,** 32138 (2016).
8. Strüber, D., Rach, S., Trautmann-Lengsfeld, S. A., Engel, A. K. & Herrmann, C. S. Antiphasic 40 Hz Oscillatory Current Stimulation Affects Bistable Motion Perception. *Brain Topogr.* **27,** 158–171 (2014).
9. Bikson, M. & Rahman, A. Origins of specificity during tDCS: anatomical, activity-selective, and input-bias mechanisms. *Front. Hum. Neurosci.* **7,** 1–5 (2013).
10. Fritsch, B. *et al.* Direct Current Stimulation Promotes BDNF-Dependent Synaptic Plasticity: Potential Implications for Motor Learning. *Neuron* **66,** 198–204 (2010).
11. Ranieri, F. *et al.* Modulation of LTP at rat hippocampal CA3-CA1 synapses by direct current stimulation. *J. Neurophysiol.* **107,** 1868–1880 (2012).
12. Pisoni, A. *et al.* Cognitive Enhancement Induced by Anodal tDCS Drives Circuit-Specific Cortical Plasticity. *Cereb. Cortex* **28,** 1132–1140 (2018).
13. Saturnino, G. B., Madsen, K. H., Siebner, H. R. & Thielscher, A. How to target inter-regional phase synchronization with dual-site Transcranial Alternating Current Stimulation. *Neuroimage* **163,** 68–80 (2017).
14. Hill, A. T., Rogasch, N. C., Fitzgerald, P. B. & Hoy, K. E. Effects of single versus dual-site High-Definition transcranial direct current stimulation (HD-tDCS) on cortical reactivity and working memory performance in healthy subjects. *Brain Stimul.* **11,** 1033–1043 (2018).
15. Alekseichuk, I. *et al.* Electric field dynamics in the brain during multi-electrode transcranial electric stimulation. *Nat. Commun.* **10,** 2573 (2019).
